# Septin remodeling is essential for the formation of cell membrane protrusions (microtentacles) in detached tumor cells

**DOI:** 10.18632/oncotarget.20805

**Published:** 2017-09-11

**Authors:** Kristine Østevold, Ana V. Meléndez, Friederike Lehmann, Gudula Schmidt, Klaus Aktories, Carsten Schwan

**Affiliations:** ^1^ Institute of Experimental and Clinical Pharmacology and Toxicology, Medical Faculty, University of Freiburg, 79104 Freiburg, Germany; ^2^ Faculty of Biology, University of Freiburg, 79104 Freiburg, Germany; ^3^ Spemann Graduate School of Biology and Medicine (SGBM), University of Freiburg, 79104 Freiburg, Germany; ^4^ Faculty of Chemistry and Pharmacy, University of Freiburg, 79104 Freiburg, Germany; ^5^ Centre for Biological Signalling Studies (BIOSS), University of Freiburg, 79104 Freiburg, Germany

**Keywords:** septin, microtentacles, microtubules, Clostridium difficile toxin, actin ADP-ribosylation

## Abstract

Microtentacles are mostly microtubule-based cell protrusions that are formed by detached tumor cells. Here, we report that the formation of tumor cell microtentacles depends on the presence and dynamics of guanine nucleotide-binding proteins of the septin family, which are part of the cytoskeleton. In matrix-attached breast, lung, prostate and pancreas cancer cells, septins are associated with the cytosolic actin cytoskeleton. Detachment of cells causes redistribution of septins to the membrane, where microtentacle formation occurs. Forchlorfenuron, which inhibits septin functions, blocks microtentacle formation. The small GTPase Cdc42 and its effector proteins Borgs regulate septins and are essential for microtentacle formation. Dominant active and inactive Cdc42 inhibit microtentacle formation indicating that the free cycling of Cdc42 between its active and inactive state is essential for septin regulation and microtentacle formation. Cell attachment and aggregation models suggest that septins play an essential role in the metastatic behavior of tumor cells.

## INTRODUCTION

Metastasis is the leading cause of death among cancer patients [1–3]. Accordingly, elucidation of the molecular mechanisms underlying cancer cell dissemination and metastasis is a main topic in cancer research. Studies from recent years have revealed that the process of metastasis is accompanied by major cytoskeletal changes, which are involved in invasion, extravasation, circulation and adhesion of metastasizing tumor cells. Especially in circulating tumor cells, cell membrane microtubule-based protrusions that extend 10 to 100 μm from the cell body are observed. These tumor cell protrusions, called microtentacles, appear to be regulated by an actin-microtubule balance [4–6]. Factors and conditions that decrease actin polymerization like cytochalasin [4] or activation of cofilin [7] increase microtentacle formation, while compounds that inhibit microtubule formation, like colchicine [4], inhibit formation of microtentacles. Importantly, these microtentacles appear to promote cell aggregation and increase the reattachment efficiency of tumor cells, thereby enhancing metastasis [4, 8, 9].

We observed that microtubule-based cell membrane protrusions are formed by bacterial protein toxins (e.g. *Clostridium difficile* toxin CDT and *C. perfringens* iota toxin) that cause ADP-ribosylation of actin in arginine 177 thereby blocking actin polymerization [10, 11]. These toxin-induced microtubule-based protrusions form a network of filaments on epithelial cells that increases bacterial adherence [10]. We also found that the protrusions contain ER membranes that are attached to microtubules via Stim1 [12]. The toxin-induced protrusions are involved in vesicle traffic and, apparently, in calcium signaling via Stim1-Orai channels. Recently, we found that the formation of the microtubule-based protrusions depends on septins [13].

Septins are guanine-nucleotide-binding cytoskeletal proteins, which form hetero-oligomeric complexes [14–17]. These complexes assemble into higher ordered structures such as filaments, bundles and rings [17–19]. The human genome contains 13 different septins, which are divided into four groups based on homology [14, 17, 20]. Septins are regulated by Cdc42, a GTP-binding protein of the Rho family [21, 22]. Previous studies showed that Cdc42 control septin functions using Borg proteins (binder of Rho GTPases, also known as Cdc42EP) as effectors [22, 23]. Recently, we have shown that the function of septins in the formation of microtubule-based cell protrusions also involve Borg proteins [13].

Septins play pivotal roles in numerous cellular functions, including cell division [24–26], branching of axons [27], vesicular traffic, exocytosis [28, 29] and cilia formation [30]. Septins are additionally involved in carcinogenesis and metastasis. For example, reports show the translocation of the mixed lineage leukemia (*MLL*) oncogene into a septin gene locus [31, 32] and in colorectal cancer, septin 9 (SEPT9) exhibits an altered pattern of isoform expression [33]. The recent introduction of a blood test with SEPT9 as an epigenetic marker for colon cancer based on the DNA methylation of the SEPT9 gene further underlines the role of SEPT9 in cancer. Given our recent finding that septins are essential for toxin-induced cell protrusion formation, we asked whether septins participate in the organization of tumor cell microtentacles. Here, we report that septins colocalize with microtubules at the base of microtentacles of tumor cells. Moreover, we show that Cdc42 and Borg proteins play a pivotal role in microtentacle formation, suggesting that toxin-induced protrusions and microtentacles share essential regulatory mechanisms. Our data additionally indicate that septins are involved in the process of metastasis.

## RESULTS

### Septin composition in breast cancer cell lines

**Figure 1:**
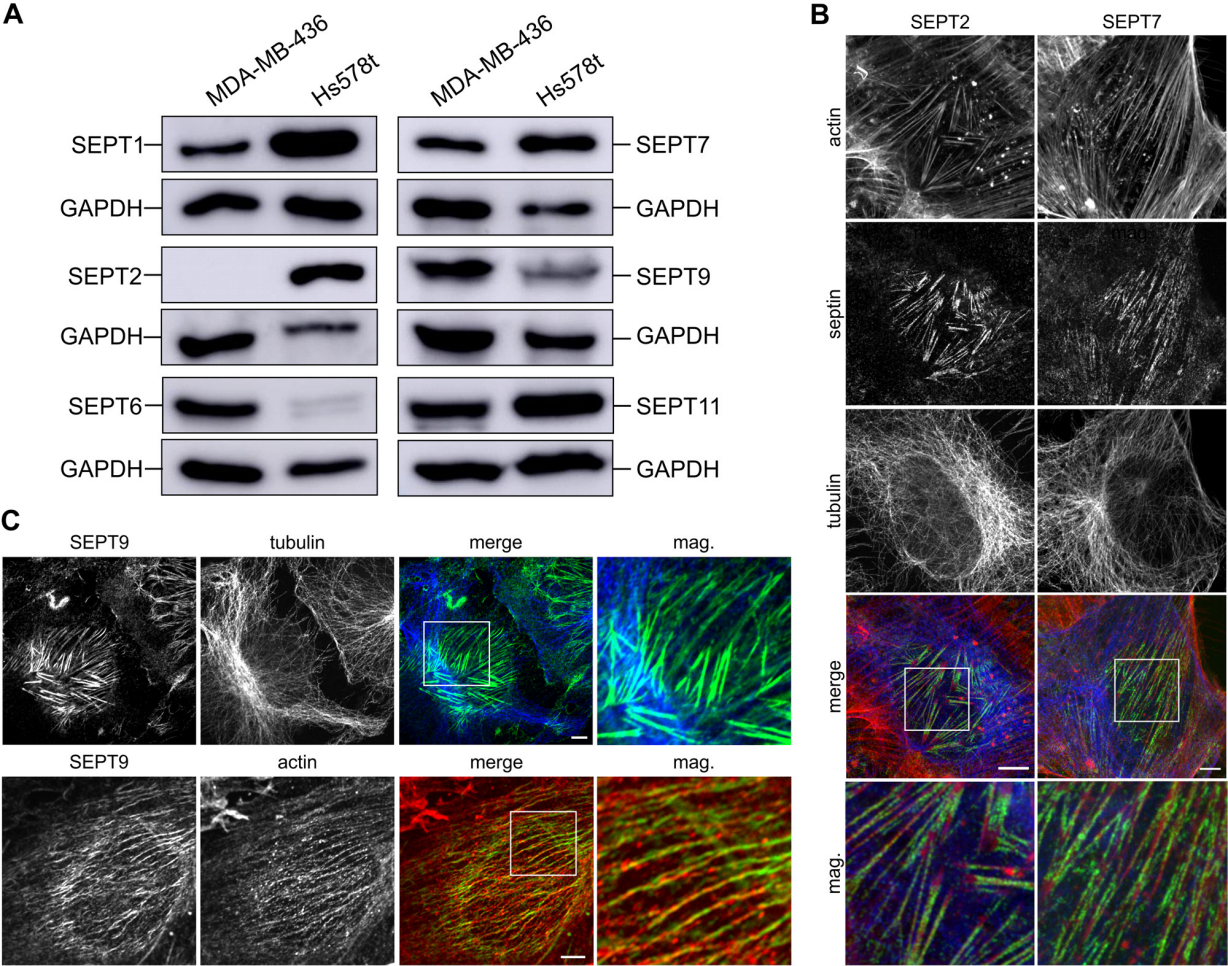
Characterization of tumor cells and septin content (**A**) Western blot of septin content in MDA-MB-436 and Hs578t whole cell lysates. (**B**) Indirect immunofluorescence of Hs578t cells for SEPT2 and SEPT7 (green), actin (red) and tubulin (blue). Septin filaments show colocalization with the actin cytoskeleton. (**C**) Indirect immunofluorescence of Hs578t cells for SEPT9 (green), actin (red) and tubulin (blue). Septin filaments show colocalization with the actin cytoskeleton. (Scale bars, 5 μm)

Because not all septins are ubiquitously expressed, we characterized septin expression in the breast cancer cell lines MDA-MB-436 and Hs578t. To this end, we analyzed the protein expression of at least one septin from each septin family using western blot analysis (Figure 1A). Both cell lines expressed SEPT7, the only unique septin lacking a paralogue, and SEPT9 from the SEPT3 group. MDA-MB-436 lacked expression of SEPT2. We therefore looked for expression of other members of the SEPT2 group and found expression of SEPT1. Hs578t lacked expression of SEPT6; thus, we confirmed the expression of SEPT11, another SEPT6 group member.

Next, we visualized the filamentous septin cytoskeleton in attached cells using immunofluorescence and transient transfection of GFP-tagged septins (Figure 1B, 1C and Supplementary Figure 1). In Hs578t cells, we found that SEPT2, SEPT7 and SEPT9 mostly colocalize with actin filaments as it was described for NIH3T3 fibroblasts in detail [18]. In MDA-MB-436 cells, we found that SEPT6, SEPT7 and SEPT9 associate with the actin cytoskeleton. By contrast, septins did not show major association with the microtubule network in either cell line under the same conditions. To explore whether microtentacle formation is unique to breast cancer cells, or whether it is a common trait of metastatic prone cancers, we additionally analyzed the septin cytoskeleton in the prostate cancer cell line LNCaP, the lung cancer cell line H1299 and the pancreas cancer cell line Capan-2. In the cell lines H1299 and Capan-2, septins colocalized with the actin cytoskeleton (Supplementary Figure 1B). In the LNCaP cells, septins rather colocalized with microtubules, since these cells lack long distinct actin filaments (Supplementary Figure 1C).

### Septins localize to the base of cancer cell microtentacles

**Figure 2:**
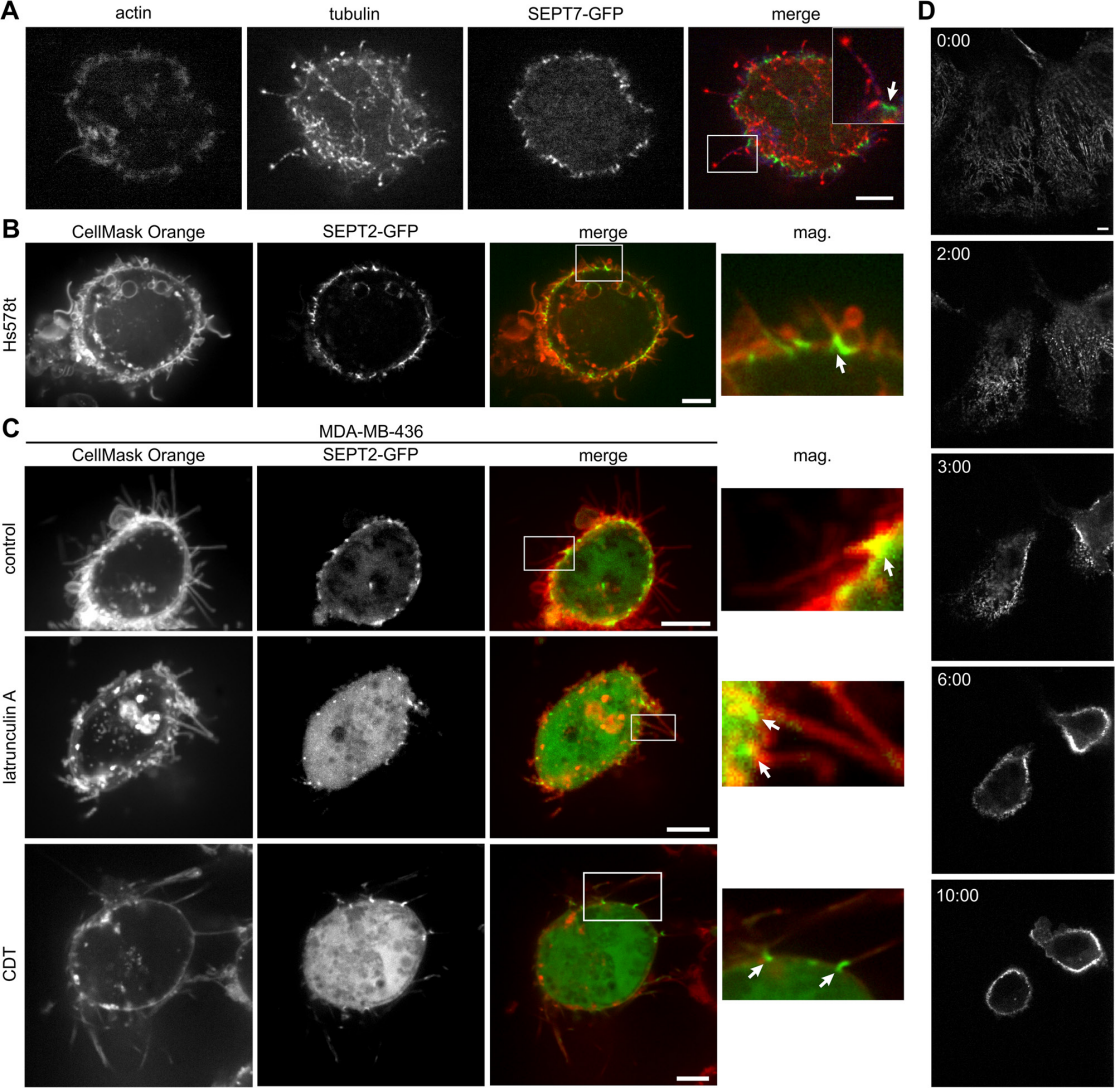
Septins localize at the base of microtentacles (**A**) Indirect immunofluorescence of MDA-MB-436 cells transfected with SEPT7-GFP for actin (blue) and tubulin (red). Cells were fixed after detachment. Microtentacles are microtubule and in some cases actin based. (**B**) Live cell confocal images of SEPT2-GFP transfected Hs578t cells. After detachment, cells were stained with CellMask Orange. Insets show the presence of SEPT2 at the base of microtentacles (arrows). (**C**) Live cell confocal images of SEPT2-GFP transfected MDA-MB-436 cells. After detachment, cells were stained with CellMask Orange. Cells were left untreated or were treated with latrunculin A (5 μM) for 30 min after detachment or with CDT (200 ng/mL CDTa and 400 ng/mL CDTb) for 1 h before detachment and during image acquisition. Insets show the presence of SEPT2 at the base of microtentacles (arrows). (**D**) Live cell confocal images of SEPT7-GFP transfected Hs578t cells. After addition of 10 mM EDTA, the cells detached and the former filamentous septin cytoskeleton rearranged and moved to the cell membrane. (Scale bars, 5 μm)

When tumor cells detach, the cytoskeleton is strongly reorganized [34–38]. Detachment disturbs the balance between microtubule expansion and actin-mediated tension. This might also involve a displacement of cortical proteins that control microtubule growth in the cell periphery. Accordingly, 15 min post detachment by trypsin/EDTA or just by complexing calcium by EDTA alone, we observed microtentacle formation in all 5 cancer cell lines (Figure 2A, 2B, 2C and Supplementary Figure 2A). MDA-MB-436 showed the most pronounced microtentacle formation with regard to both length and number. Immunofluorescence studies revealed that the longer microtentacles are microtubule based, but also contain actin to some degree (Figure 2A). Shorter structures that only have actin as structural component and resemble the morphology of filopodia were also formed.

**Figure 3:**
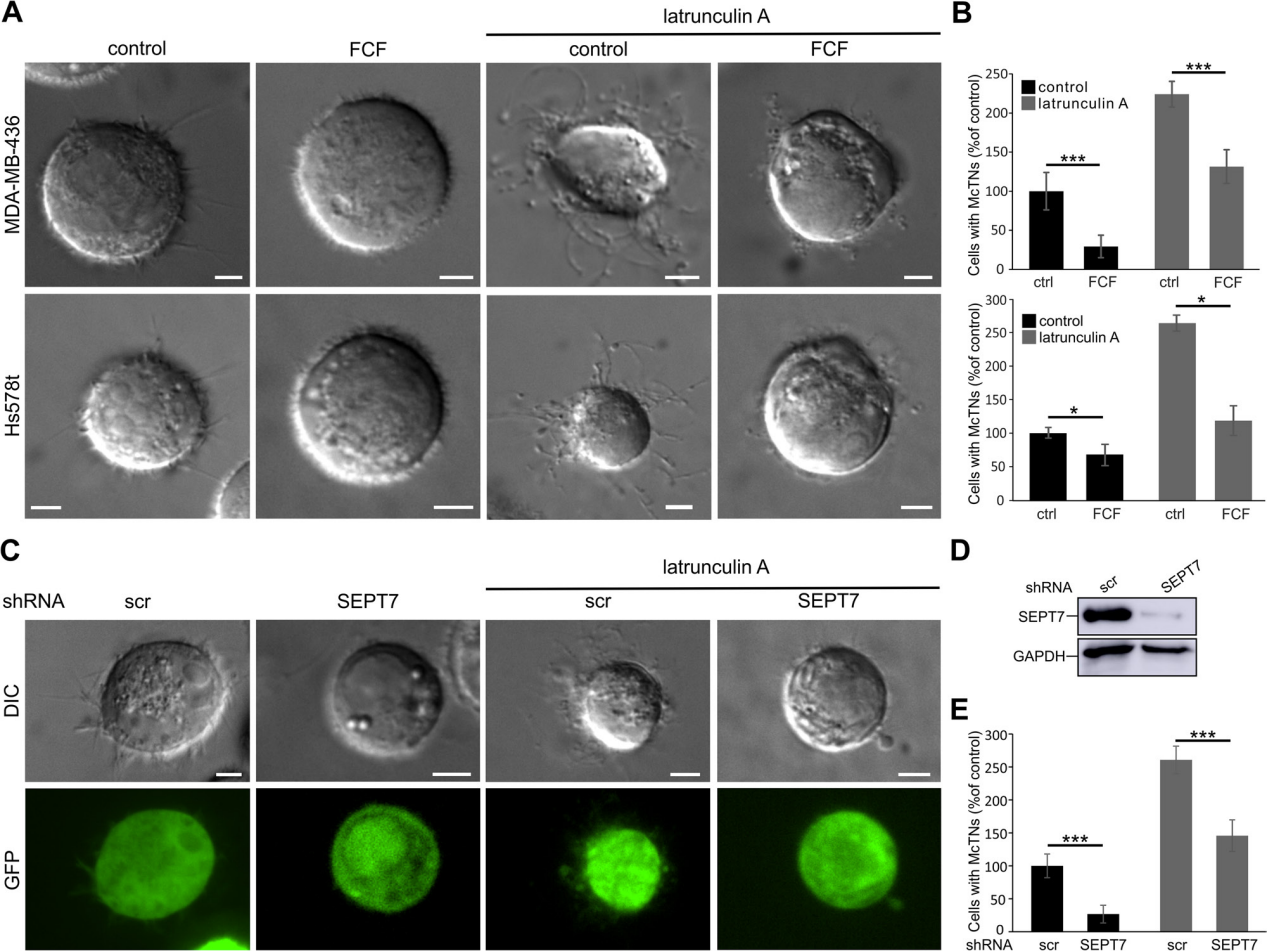
Septins influence microtentacle formation (**A**) Live cell differential interference contrast (DIC) images of MDA-MB-436 and Hs578t cells. Cells were treated with FCF (50 μM) or DMSO as solvent control for 3 h prior to detachment and during image acquisition. After detachment, cells were treated with latrunculin A (5 μM) for 30 min. Latrunculin A increased microtentacle formation while FCF inhibited the process. (**B**) MDA-MB-436 and Hs578t cells were treated as in A. Microtentacle formation was quantified after detachment. Data are given ±SEM, ≥150 cells were scored, *n* = 5. (**C**) DIC and confocal images of control shRNA (scr) and SEPT7 shRNA transfected MDA-MB-436 cells after detachment and 30 min treatment with latrunculin A (5 μM). Knock-down of SEPT7 caused a decrease in microtentacle formation. Treatment with latrunculin A increased microtentacle formation both in control and knock-down cells. (**D**) Western blot for SEPT7 of MDA-MB-436 cell lysate after knock-down of SEPT7 by shRNA. (**E**) MDA-MB-436 cells were treated as in C. Microtentacle formation was quantified after detachment. Data are given ±SEM, ≥100 cells were scored, *n* = 6. (Scale bars, 5 μm)

Furthermore, we observed that the formation of microtentacles was accompanied by reorganization of the septin cytoskeleton (Figure 2 and Supplementary Figure 2A and Supplementary Movie 1). The disassembly of septin filaments was followed by the translocation of septins to the cell membrane, where they were localized at the base of microtentacles. Recently, we observed similar structures after treatment of epithelial cells with the ADP-ribosylating actin-depolymerizing toxin CDT [13]. When MDA-MB-436 cells were additionally treated with CDT or actin-depolymerizing latrunculin A, microtentacle formation with septins at the base was enhanced (Figure 2C, 3B and Supplementary Figure 2B). Worth mentioning, we also observed that emerging microtentacles of cancer cells contained endoplasmic reticulum (ER) (Supplementary Figure 3 and Supplementary Movies 2, 3) similar as reported for CDT-induced protrusions of adherent cells [12].

### Septins are essential for microtentacle formation

To characterize the functional role of septins in cell detachment-induced microtentacle formation, we employed forchlorfenuron (FCF), which inhibits septin dynamics [39]. After treatment with FCF (50 μM) for 3 h, MDA-MB-436 and Hs578t cells were detached and the number of cells forming microtentacles longer than the radius of the cell body was quantified (Figure 3A, 3B). In MDA-MB-436 cells, FCF inhibited microtentacle formation by ~75%. In Hs578t cells, the effect of FCF was not as strong, but significant with a decrease in microtentacle formation of ~25%.

Treatment of both cell lines with latrunculin A (5 μM) for 30 min caused more than a doubling in microtentacle positive cells (Figure 3A, 3B). Also under these conditions, FCF caused a strong decrease in microtentacle formation. To confirm that the FCF-induced decrease in microtentacle formation was caused by the inhibition of septin dynamics, we additionally studied microtentacle formation after shRNA knock-down of SEPT7 (Figure 3C, 3D). The SEPT7 knock-down reduced microtentacle formation by ~75% when compared to transfection with a non-targeting shRNA (Figure 3E). Also in this case, latrunculin A (5 μM) treatment nearly doubled the number of microtentacle-positive cells. However, knock-down of SEPT7 still reduced microtentacle formation by ~40%.

### Septin mediated microtentacle formation is dependent on Cdc42 and its effector proteins Borgs

**Figure 4:**
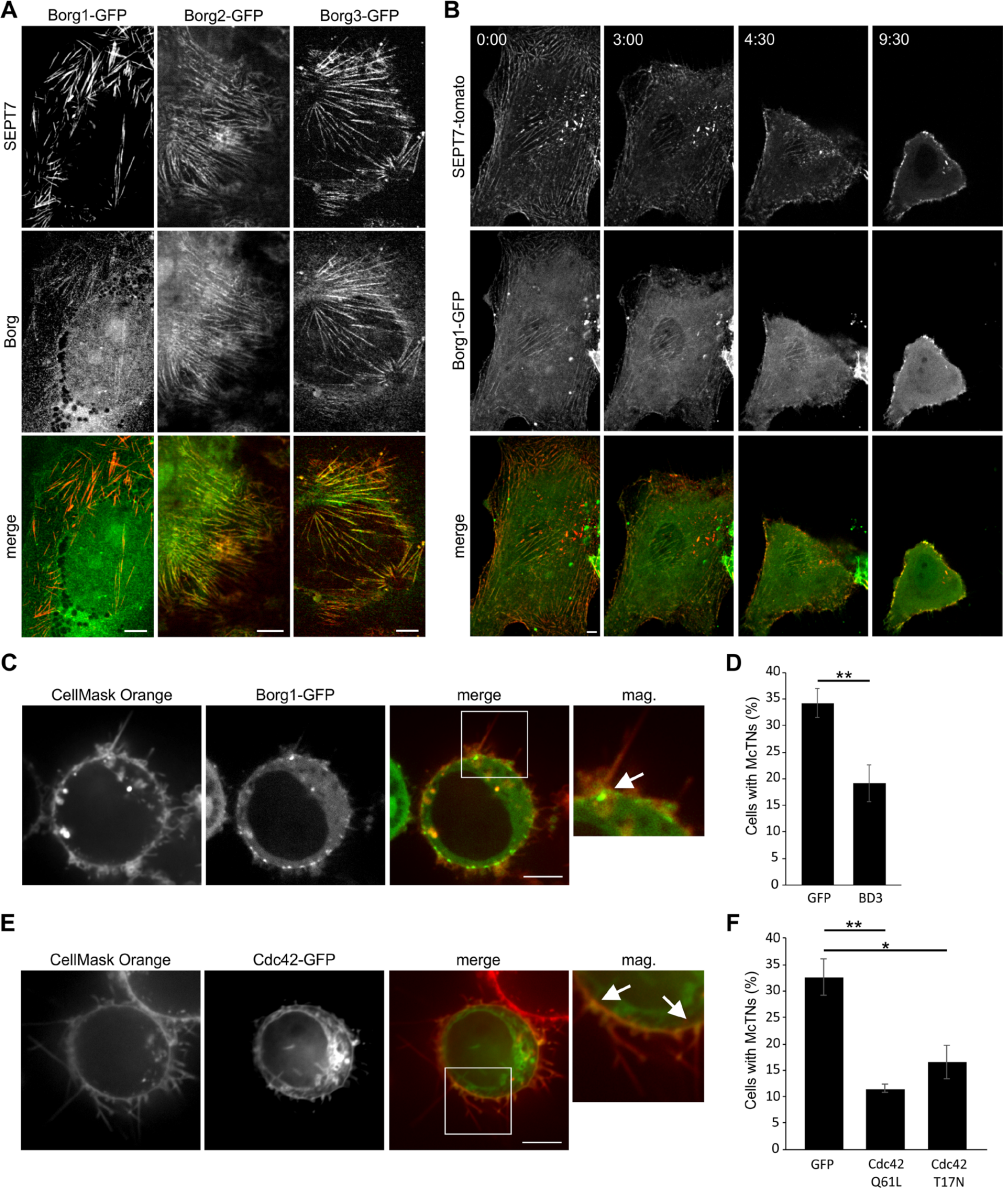
Cdc42 and Borgs regulate septins during the formation of microtentacles (**A**) Indirect immunofluorescence of Hs578t cells transfected with Borg 1-, 2-, or 3-GFP and stained for SEPT7 (red). Septin filaments show colocalization with Borgs. (**B**) Live cell confocal images of SEPT7-tomato and Borg1-GFP transfected Hs578t cells. After addition of 10 mM EDTA, the cells were detached and both septin and Borg moved to the cell membrane. (**C**) Live cell confocal images of Borg1-GFP transfected MDA-MB-436 cells. After detachment, cells were stained with CellMask Orange. Borg1 can be seen at the base of microtentacles (arrow). (**D**) MDA-MB-436 cells were transfected with BD3-GFP or GFP as a control. The formation of microtentacles was quantified after detachment. Cells transfected with BD3 showed decreased microtentacle formation. Data are given ± SEM, ≥ 80 cells were scored, *n* = 4. (**E**) Live cell confocal images of Cdc42-GFP transfected MDA-MB-436 cells. After detachment, cells were stained with CellMask Orange. Cdc42 can be seen at the base of microtentacles (arrows). (**F**) MDA-MB-436 cells were transfected with dominant active Cdc42 (Cdc42Q61L), dominant negative Cdc42 (Cdc42T17N) or GFP as a control. The formation of microtentacles was quantified after detachment. Cells transfected with dominant active or dominant negative Cdc42 showed decreased microtentacle formation. Data are given ± SEM, ≥100 cells were scored, *n* = 3. (Scale bars, 5 μm)

The Rho GTPase Cdc42 and its effector proteins Borgs are involved in regulation of septins [22]. Previous studies showed that Cdc42 and Borgs regulate protrusion formation induced by actin-depolymerizing toxins [13] and thus we were interested to see whether the same mechanism is employed in microtentacle formation in cancer cells. We observed that Borgs 1, -2 and -3 colocalized with both SEPT2 and SEPT7 filaments in attached Hs578t cells (Figure 4A and Supplementary Figure 4A) and in attached MDA-MB-436 cells, Borgs 1, -2 and -3 were found to colocalize with SEPT9 (Supplementary Figure 4A). When cells were detached, septins (Figure 2A, 2B, 2C) and Borgs (Figure 4B, 4C, Supplementary Figure 4B and Supplementary Movie 4) were localized at the base of microtentacles. We additionally observed the presence of Cdc42 at the base of microtentacles (Figure 4E and Supplementary Figure 4B).

Next, we analyzed the role of Borgs in the formation of microtentacles by overexpression of the septin-binding domain BD3 of Borg3 in MDA-MB-436 cells. The BD3 fragment is not controlled by Cdc42. In contrast, the BD3 fragment causes sequestration of septins [18]. Quantification of cells forming microtentacles after detachment revealed that the overexpression of the BD3 domain inhibited microtentacle formation by ~40% (Figure 4D).

To confirm the essential role of Cdc42 in microtentacle formation, we overexpressed dominant active and dominant negative Cdc42 in MDA-MB-436 cells. Under both conditions (over-expression of dominant active or inactive Cdc42), the formation of microtentacles was significantly reduced (Figure 4F). These results are very similar to recent findings obtained with dominant active and dominant negative Cdc42 in toxin-induced protrusion formation [13], indicating a crucial role of Cdc42 cycling between its inactive (GDP bound) and active (GTP bound) forms in septin organization.

### Septins aid microtentacle formation through interaction with EB1

**Figure 5:**
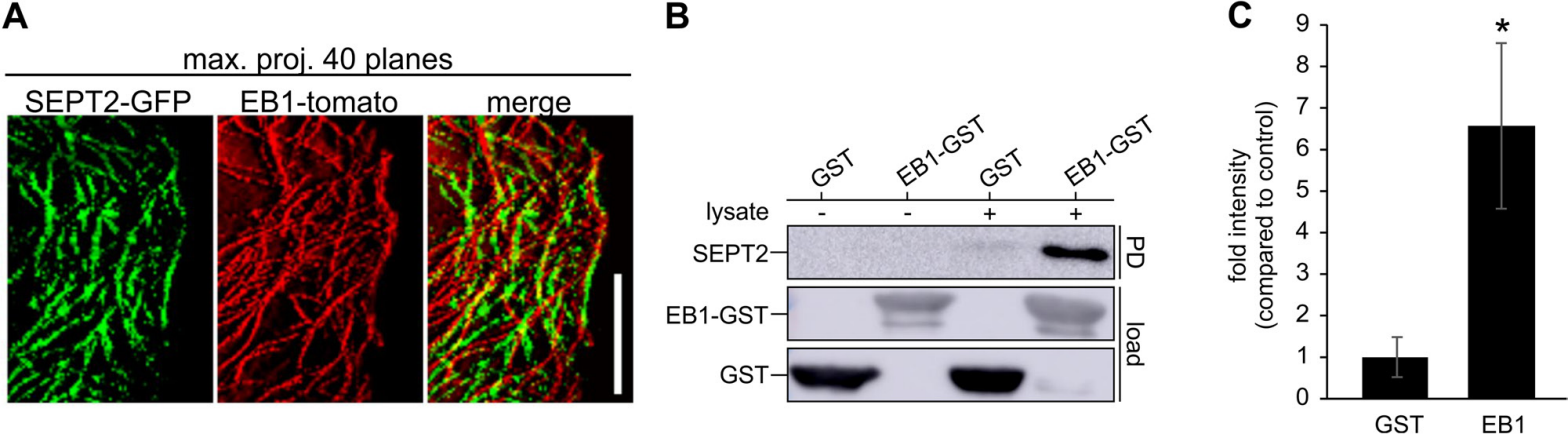
Septins interact with EB1 in cancer cells (**A**) Hs578t cells were transfected with EB1-tomato and SEPT2-GFP. Maximal projection of 40 planes from a timelapse movie with 3 s interval of attached cells shows the tracks of the EB1 comets (red) and SEPT2 filaments (green). EB1 tracks are coaligned with septin filaments. (Scale bar, 5 μm) (**B**) Representative blot of an EB1-GST pull-down of endogenous SEPT2 from Hs578t lysate. GST-loaded beads were used as a control. (**C**) Quantification of blots as in B. Blots were normalized to the load. Data are ± SEM, *n* = 6.

Septins guide the growing end of microtubules into toxin-induced protrusions through the interaction with the microtubule binding protein EB1 [13]. We showed a close association of septins and EB1 over time in breast cancer cells using SEPT2-GFP and EB1-tomato transfected Hs578t cells (Figure 5A). Maximum projection of sequential EB1-tomato images visualizes EB1 localized at the tip of polymerizing microtubules moving along septin filaments in attached cells. Pull-down experiments, using whole cell lysates and EB1-GST beads, additionally confirmed an interaction between EB1 and SEPT2 in Hs578t cells (Figure 5B, 5C). We furthermore confirmed the interaction between EB1 and SEPT7 in MDA-MB-436 cells (Supplementary Figure 5). Consequently, septins might function as a structural link between the membrane and microtubules during the formation of microtentacles.

### Septins are important for efficient tumor cell reattachment and aggregation

**Figure 6:**
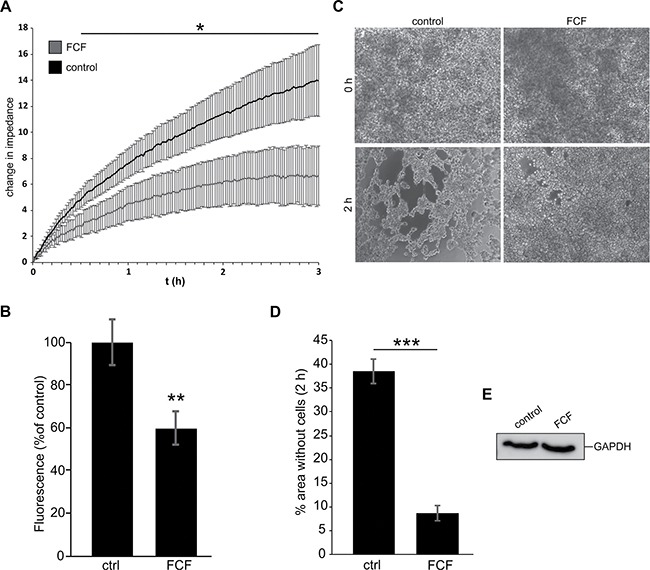
Inhibition of septin dynamics leads to decreased reattachment efficiency and cell aggregation (**A**) Real time changes in impedance caused by cell reattachment. MDA-MB-436 cells were treated for 3 h with FCF (50 μM) or DMSO as a solvent control. FCF treated cells showed decreased reattachment efficiency compared with control cells. The graph represents mean ±SD for each treatment group, *n* = 3. (**B**) Fluorescence of DAPI-labeled reattached MDA-MB-436 cells. Cells were treated with FCF (50 μM) or DMSO for 3 h prior to detachment, and during the experiment. After detachment, cells were added to a fibronectin-coated 96 well plate and placed on a shaker for 1 h. FCF-treated cells showed decreased reattachment efficiency. Data are ±SEM, *n* = 5. (**C**) Hanging drop cell aggregation assay. MDA-MB-436 cells were treated with FCF (50 μM) or DMSO for 3 h and during the experiment. After detachment, cell suspension drops were deposited on the inner side of a cell culture dish lid. The lid was turned and the cells were incubated for 2 h. FCF-treated cells show decreased cell aggregation. (**D**) Cells treated as in C. Quantification of cell-free areas in the culture medium drop as a percentage of the whole image. DMSO-treated cells have significantly more open areas compared to the FCF-treated cells. Data are ± SEM, *n* = 3. (**E**) Cells from C were collected after the experiment and lysed before analysis of GAPDH levels by western blot. Blot shows similar GAPDH amounts in both treatment groups.

Previous studies suggest that microtentacles promote cell aggregation and facilitate reattachment of circulating tumor cells [4]. Because septins play important roles in microtentacle formation, we analyzed whether FCF treatment would alter the reattachment efficiency of cancer cells. Using electric cell impedance sensing (ECIS), we observed that FCF treatment caused a significant decrease in the reattachment efficiency of MDA-MB-436 cells (Figure 6A). Incubation of detached MDA-MB-436 cells on fibronectin-coated plates on a shaker exhibited a similar decrease (~40%) in reattachment efficiency after FCF-treatment (Figure 6B).

Circulating tumor cell reattachment and metastasis efficiency are influenced by cell aggregation. Circulating tumor cell aggregates introduced experimentally into the blood stream are reportedly far more efficient than individual carcinoma cells in seeding metastatic colonies [40]. Using the hanging-drop cell assay, we studied the aggregation efficiency of detached MDA-MD-436 cells over the course of 2 h using light microscopy (Supplementary Movie 5). During this time, control cells clustered together, exposing free areas in the culture medium drop. Cells treated with FCF (50 μM) exhibited a significant decrease in cell aggregation (Figure 6C, 6D). A loss of cells, for example by anoikis, was excluded by subsequent lysis of the cells and western blot analysis of GAPDH (Figure 6E). Together, these findings suggest that septin dynamics play a pivotal role in cancer metastasis.

## DISCUSSION

Long cell protrusions, named microtentacles, appear to be a common trait of circulating tumor cells, and are suggested to be the result of changes of the balance between actin and microtubules [4–6, 36]. Actin-depolymerizing toxins like the binary actin-ADP-ribosylating toxin CDT induce microtubule-based cell protrusions, which share structural features with microtentacles [10, 11]. Recently, we reported that toxin-induced protrusions depend on septin restructuring at the cell membrane [13]. Therefore, we were tempted to study the role of septins in microtentacle formation in tumor cells. We observed that different types of tumor cells possess different profiles of septin proteins. However, the various septin proteins detected covered at least one isoform from each of the 4 septin subgroups. We observed that septins are located close to the base of the protrusions. Several findings indicate that septins are essential for microtentacle formation. First, alteration of septin dynamics by forchlorfenuron (FCF) strongly inhibited microtentacle formation. At 50 μM FCF, microtentacle formation was reduced by 25–75%, depending on the cell line. Second, we found that the formation of microtentacles depended on the Rho GTPase Cdc42 and its effectors Borg proteins. We observed colocalization of Cdc42, Borg and septins at the base of microtentacles. Moreover, as reported for a functional Cdc42-septin interaction in other systems [13, 19, 41], cycling between an active and inactive state of Cdc42 appears to be necessary for formation of microtentacles, because overexpression of dominant active or inactive Cdc42 inhibited microtentacle formation. Finally, overexpression of the septin-interacting domain BD3 of Borg significantly reduced protrusion formation. Thus, a similar molecular orchestration of interacting proteins appear to be involved in microtentacle formation as reported for toxin-induced protrusions [13]. Moreover, as observed for the toxin-induced microtubule-based protrusions [13], the functional connection between septins and microtubules appear to depend on an interaction of septin molecules with EB proteins at the plus end of microtubules.

Various septin proteins have been associated with carcinogenesis and metastasis. This is especially true for SEPT9. In human acute myeloid leukemia, the gene of SEPT9 (*MSF*) is a fusion partner of mixed lineage leukemia gene MLL [31, 42]. SEPT9 is frequently overexpressed in breast cancer cell lines and breast cancer tissue [43, 44]. Overexpression also occurs in prostate cancer [45, 46], ovarian cancer [47] and in head and neck squamous cell carcinoma [48]. Furthermore, it is shown that aberrant methylation in the promoter region of SEPT9 is associated with colorectal cancer [49, 50]. In 2016, the DNA-methylation based biomarker SEPT9 became the first blood-based colorectal cancer-screening test approved by the FDA.

Moreover, SEPT9 appears to be essential for pseudopodial actin dynamics and control of epithelial to mesenchymal transition (EMT) [51], a phenomenon suggested to be crucial for invasion and metastasis [36, 52, 53]. Studies on the precise role of SEPT9 are hampered by the fact that various isoforms of SEPT9 are expressed. However, the majority of SEPT9 isoforms studied was shown to strongly increase migration of tumor cells [44]. In addition, other septins appear to play a role in carcinogenesis. For example, in the squamous cell carcinoma cell line DJM-1 depletion of SEPT1 inhibited cell spreading [54] and in the invasive breast cancer cell line MDA-MB-231, depletion of either SEPT2 or SEPT7 inhibited cell migration and invasion [55]. Moreover, septins, Cdc42 and Borg proteins play a crucial role in cancer-associated fibroblasts (CAFs), which are non-cancerous cells present in solid tumors that remodel the tumor matrix and promote cancer invasion and angiogenesis [56].

While several studies showed that septins are involved in cell migration [55, 57, 58], their precise roles are not entirely clear. It has been suggested that the ability of septins to interact with membranes, as well as with the actin and microtubule cytoskeletons favors these proteins as scaffolds for organization of functional complexes localized at the cell cortex, such as leading edge of migrating cells, neuronal growth cones or - in our case - formation of microtubule-based cell protrusions [16].

The data presented indicate that microtentacles and toxin-induced protrusions share many basic structural and functional features. This is in agreement with our view that toxin-induced protrusions are not only artefacts of toxin treatment but are specific physiological responses of the cytoskeleton towards defined changes in the homeostasis of cytoskeletal factors, especially concerning the actin cytoskeleton. These changes in the balance of the interplay of cytoskeletal components might be particularly relevant in cells leaving their usual environment. Accordingly, we found that septins are mainly localized to the actin cytoskeleton in adherent cells. When tumor cells are detached, which might mirror cell release from their usual environment, changes in the actin cytoskeleton occur. First, actin filaments start to contract; subsequently the density of cortical actin close to the plasma membrane is increased, while actin density in the center of the cell is reduced. These changes are accompanied by redistribution of septins to the membrane. This process appears to be key for protrusion formation. Whether Cdc42 and Borgs are involved in the redistribution to the membrane or play an essential role in the local organization of the septin structures at the membrane, or both, remains to be clarified.

The precise role of microtentacles in processes related to metastasis e.g., invasion, extravasation, migration or adhesion is still enigmatic. We propose that septins play major parts in these processes, because their restructuring is a prerequisite in microtentacle formation. To verify functions of septins in tumor-matrix and cell-cell interaction, we studied the effects of FCF on reattachment of previously detached tumor cells. We show that FCF potently inhibits the reattachment of tumor cells. Moreover, we tested the effects of FCF on tumor cell aggregation in the hanging drop model. Here, we observed that the septin inhibitor effectively decreased tumor cell aggregation. While we cannot definitely say that this is caused by inhibition of microtentacle formation, the findings clearly indicate that septins are involved in attachment and cell-cell aggregation. Our findings suggest that septins could be a possible novel target for breast cancer therapy.

The similarity of structural and functional features of microtentacles and toxin-induced membrane protrusions allows us to take toxin-induced protrusions as a model for microtentacles. The toxin model has many experimental advantages including high structural uniformity and stability. Moreover, formation of protrusions induced by CDT is very reliable. Therefore, the actin ADP-ribosylating toxin(s) are highly instrumental to study tumor cell microtentacles and their functions. In this respect, it is of interest that toxin-based protrusions contain ER structures, which are connected to microtubules via Stim-1 [12]. On the other hand, Stim1 interacts in the protrusions with Orai channels involved in store-operated calcium entry (SOCE). Preliminary data indicate that this is also true for microtentacles. It would be of great interest to study whether calcium signaling occurs in microtentacle possibly via Stim1 and Orai channels, because a connection between septins and SOCE has been reported [59, 60].

## MATERIALS AND METHODS

### Cell culture and transient transfections

MDA-MB-436 (kindly provided by Prof. Tilman Brummer, authentication by Eurofins Medigenomix Forensik, Ebersberg, Germany) and Hs578t (kindly provided by Markus Jäger, authentication by Eurofins Medigenomix Forensik, Ebersberg, Germany) cells were cultured in DMEM/F12 supplemented with 10% fetal calf serum (FCS) and 1% penicillin streptomycin. LNCaP (DSMZ, Braunschweig, Germany) and Capan-2 (ATCC, Wesel, Germany) cells were cultured in RPMI supplemented with 10% and 15% FCS respectively, and 1% penicillin streptomycin. H1299 (BIOSS Toolbox, Freiburg, Germany) cells were cultured in DMEM supplemented with 10% FCF, 1% penicillin streptomycin, 1% non-essential amino acids and 1% sodium pyruvate. Subconfluent cells were cultured for 1–4 days. Mycoplasma contamination was excluded by monthly testing. Cells were transfected using Lipofectamine 3000 (Invitrogen, Darmstadt, Germany).

For immunostainings, cells were seeded on HCl-washed coverslips or on PEI (25 μg/mL in 150 mM NaCl) coated μ-Slide 8 wells (Ibidi, Martinsried, Germany) for detached cells. For live-cell imaging, cells were seeded on glass-bottom dishes (Greiner Bio-One, Frickenhausen, Germany).

Overexpression of proteins was done by transient transfection. The desired protein was fluorescently labelled using the plasmids pEGFP-N1 and ptdTomato-N1 from Clonetech (Mountain View, CA, USA) and overexpression was confirmed by microscopy.

For FCF treatment, cells were kept in DMEM/F12 medium without FCS. Control cells were treated with an equal volume DMSO, which never exceeded 0.05%.

### Expression and purification of proteins

GST and GST-EB1 were produced in *Escherichia coli* BL21 from the pGEX 4T vector. After induction with isopropyl β-d-1-thiogalactopyranoside (IPTG), bacteria were incubated for 4 h at 37 °C. Bacteria were lysed in 50 mM Tris·HCl (pH 7.5), 150 mM NaCl, 5 mM MgCl_2_, 10% glycerol, and 0.5% Triton X-100 by sonication, and the cleared lysates were incubated with glutathione-Sepharose 4B (GE Healthcare, Munich, Germany) beads for 90 min at 4°C. The beads were washed one time in lysis buffer and three times with 50 mM Tris·HCl (pH 7.5), 100 mM NaCl, 2 mM MgCl_2_, 10% glycerol, and 1% Nonidet P-40.

### shRNAs

The used shRNAs were cloned into the pSUPER.retro.gfp+neo vector (Oligoengine, Seattle, WA, USA). The following already published target sequences were used: SEPT7 CTTGCAGCTGTGACTTATA [61] and control GATCTGATCGACACTGTAA.

### Antibodies, fluorescent dyes, and fluorescent proteins

Mouse monoclonal anti–α-tubulin (200 μg/mL), polyclonal rabbit anti-SEPT2 (100 μg/mL), polyclonal rabbit anti-SEPT7 (50 μg/mL), polyclonal rabbit anti-SEPT9 (300 μg/mL) and polyclonal anti-SEPT11 (100 μg/mL) were purchased from Sigma Aldrich (Taufkirchen, Germany). Mouse anti-GAPDH (1 mg/mL) was from Millipore (Darmstadt, Germany). Mouse monoclonal anti-GST (200 μg/mL), rabbit polyclonal anti-SEPT6 (200 μg/mL), mouse monoclonal anti-SEPT1 (200 μg/mL) were from Santa Cruz (Heidelberg, Germany). Secondary Alexa568- and Alexa488-conjugated antibodies (2 mg/mL) were from Invitrogen (Darmstadt, Germany). Secondary CF405M-conjugated antibodies (2 mg/mL) and CF405M-labeled phalloidin were from Biotrend (Cologne, Germany). Mouse anti-actin antibody (1 mg/mL) and atto565-labeled phalloidin were from Hypermol (Bielefeld, Germany). CellMask Orange plasma membrane stain was purchased from Thermo Fisher Scientific (Darmstadt, Germany). Peroxidase-conjugated secondary antibodies against mouse (1 mg/mL) and rabbit (92 μg/mL) were from Rockland Antibodies and Assays (Limerick, PA, USA) and Cell Signaling Technologies (Leiden, Netherlands), respectively. For immunostainings, both primary, secondary antibodies and atto565-labeled phalloidin were diluted 1:400. CF405M-labeled phalloidin was diluted 1:200. For western blots, primary antibodies were diluted 1:2000 with the exception of GAPDH, which was diluted 1:20,000. Secondary antibodies were diluted 1:3000.

### Immunostaining

Cells were fixed for 10 min with 4% paraformaldehyde in PBS, permeabilized (10 min) with 0.15% Triton X-100 in PBS, and blocked with 1% BSA in PBS or 10% normal goat serum (Life Technologies, Darmstadt, Germany) for 30 min. Incubation with the primary antibody was for 90 min at RT. Cells were incubated with the suitable secondary antibody for 1 h. Finally, cells were dried and embedded with FluoProtect (Hypermol, Bielefeld, Germany) or with Mowiol supplemented with DABCO (Sigma, St. Louis, MO, USA).

Detached cells were fixed by addition of 8% warm paraformaldehyde to the cell culture medium (final 4% paraformaldehyde) and was left in PBS after the staining procedure to preserve microtentacles.

Cells were analyzed with an Axiovert 200M microscope (Carl Zeiss, Jena, Germany), driven by Visiview (Visitron, Puchheim, Germany) imaging software with plan-apochromat objectives, a Yokogawa CSU-X1 spinning disk confocal head with emission filter wheel, and a Coolsnap HQ II digital camera with 405-, 488-, and 561-nm laser lines. Images were processed with Metamorph software.

### Live-cell confocal imaging

For live-cell imaging, cells were incubated in a chamber with humidified atmosphere (6.5% CO_2_ and 9% O_2_) at 37 °C on the microscope mentioned in the immunostaining section. For quantification of microtentacle formation, cells with mictrotentacles extending greater than the radius of the cell body were scored as positive.

### Pull-down assays

Cells were lysed in 50 mM Tris·HCl (pH 7.5), 100 mM NaCl, 2 mM MgCl_2_, 10% glycerol, 1% Nonidet P-40 and 1 mM PMSF. Lysis was increased by passing the cells through a syringe with a 26-G needle. Lysates were cleared by centrifugation at 17,000 × *g*. Lysates were incubated with GST or GST-EB1 bound to glutathione-Sepharose 4B (GE Healthcare) for 1.5 h at 4°C. Beads were washed three times with lysis buffer. The samples were subjected to western blot analysis. The blots were analyzed using Multi Gauge V3.0.

### Quantification of cell reattachment by electric cell-substrate impedance sensing (ECIS)

Cells were treated for 3 h with FCF (50 μM) or DMSO. After detachment, 3.5 ×10^5^ cells were seeded on a poly-HEMA precoated dish. After 15 min incubation, 500 μL of cell suspension was added in triplicate to the ECIS microwell (Applied Biophysics, Troy, NY, USA). The ECIS analyzer measured cell reattachment as perturbation to the flow of current passing through the plate. Readings were taken every 80 sec for 3 h. The data was collected with the ECIS software VERSION v.1.2.201.0 PC. Cell reattachment efficiency was calculated as change of impedance over time.

### Cell reattachment assay

Cells were treated with FCF (50 μM) or DMSO for 3 h prior to detachment and during the experiment. After detachment, cells were added to a fibronectin coated (10 μg/mL) 96 well plate and placed on a shaker for 1 h. Medium was aspirated and the attached cells were fixed for 10 min with 4% paraformaldehyde in PBS, washed and stained with DAPI before analysis with a TECAN plate reader (excitation 360 nm/emission 460 nm).

### Hanging drop cell aggregation assay

Cells were treated with FCF (50 μM) or DMSO for 3 h prior to detachment and during the experiment. Cell suspensions of 1.5 x10^6^ cells/mL were prepared and drops of 30 μL were carefully deposited on the inside of a cell culture dish lid. The lid was turned and placed on top of the plate filled with PBS. Pictures were taken at the beginning of the experiment and after 2 h of incubation. The area of the images not containing cells was measured using Metamorph software. A higher area without cells was interpreted as a higher degree of cell aggregation. After the experiment, the drops were collected and GAPDH contents were analyzed by western blot to ensure equal cell number in the two treatment groups. To study the process of aggregation over time, cell drops of 50 μL were added to GravityPLUS Hanging Drop System 96 well plates (InSphero, Schlieren, Switzerland) and images were taken every 3 min for 2 h using the Lionheart FX automated microscope (BioTek, Bad Friedrichshall, Germany).

### Statistics

Student's *t* test was applied when two groups with normal distribution had to be compared. The Mann-Whitney *U* test was used for data without normal distribution. For comparison of more than two groups, ANOVA was applied. Statistics evaluation was performed with the Sigma Stat software (Jandel Scientific). *P* values < 0.05 were considered statistically significant and marked with an asterisk (*P < 0.05; **P < 0.01; ***P < 0.005).

## SUPPLEMENTARY MATERIALS FIGURES












