# Supplementary material for: Septin remodeling is essential for the formation of cell membrane protrusions (microtentacles) in detached tumor cells
Source: Oncotarget. 2017 Sep 11;8(44):76686–98. doi: 10.18632/oncotarget.20805 (PMC5652735; doi:10.18632/oncotarget.20805)
Supplement: Supplementary file 1 [file oncotarget-08-76686-s001.pdf]

Septin remodeling is essential for the formation of cell membrane protrusions (microtentacles) in detached tumor cells

SUPPLEMENTARY MATERIALS

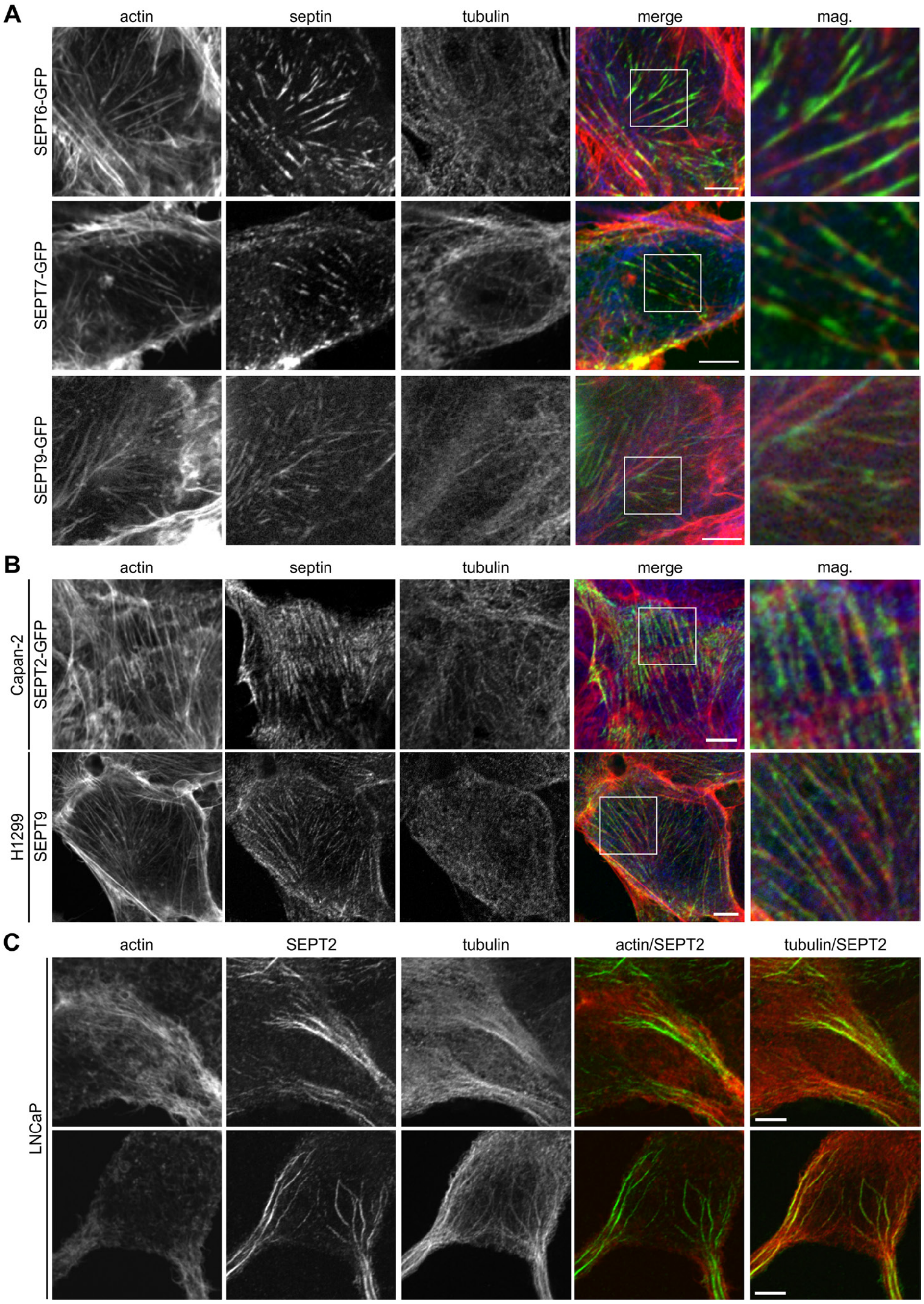

Supplementary Figure 1: Characterization of tumor cells and septin content. (**A**) Indirect immunofluorescence of MDA-MB-436 cells transfected with SEPT6-, 7-, or 9-GFP for actin (red) and tubulin (blue). Septin filaments show colocalization with the actin cytoskeleton. (**B**) Indirect immunofluorescence of Capan-2 cells transfected with SEPT2-GFP for actin (red) and tubulin (blue) and H1299 cells for SEPT9 (green), actin (red) and tubulin (blue). Septin filaments show colocalization with the actin cytoskeleton. (**C**) Indirect immunofluorescence of LNCaP cells for SEPT2 (green), actin (red) and tubulin (red). Septin filaments partially colocalize with both the actin cytoskeleton and with microtubules. (Scale bars, 5 µm)

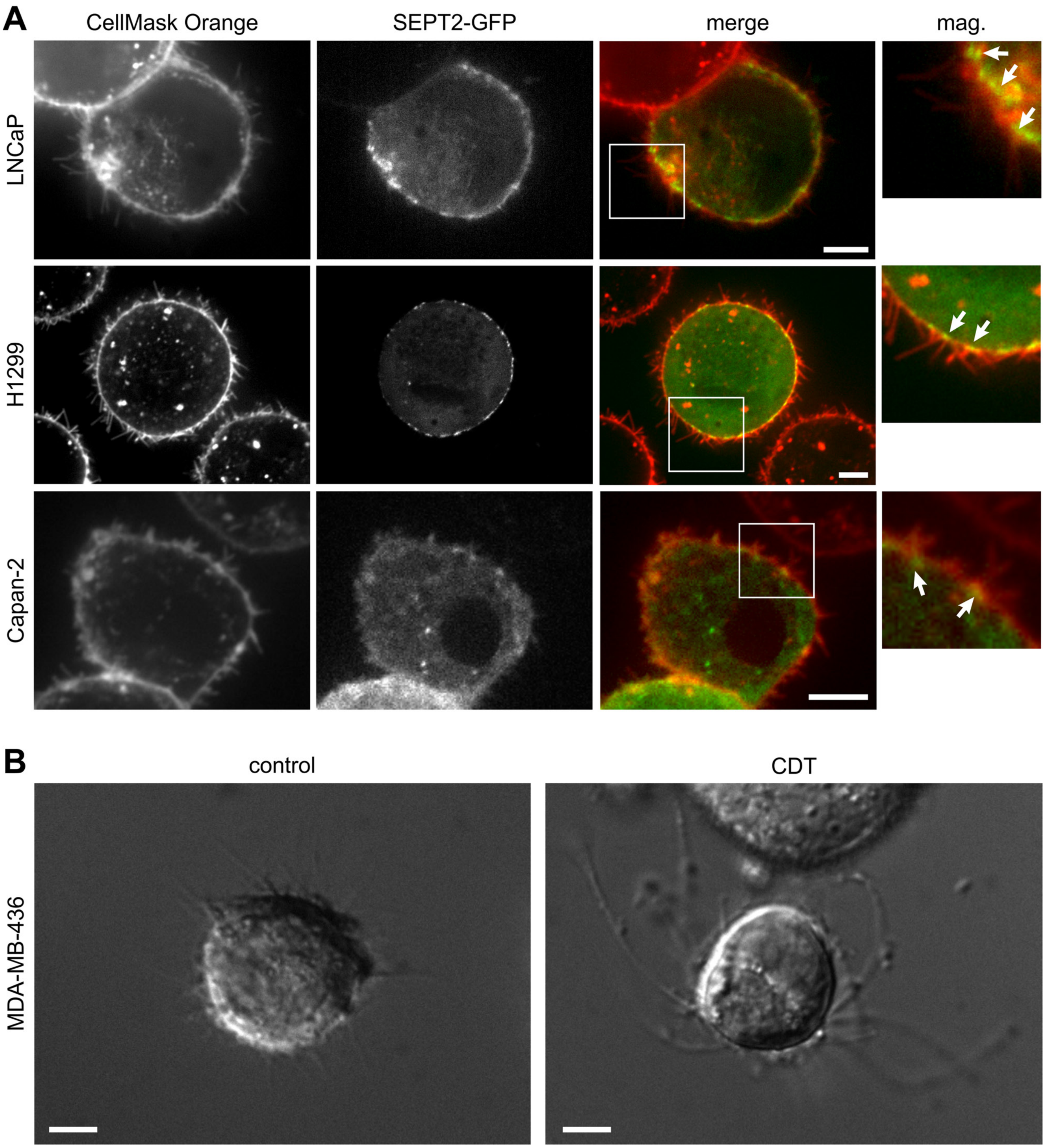

Supplementary Figure 2: Septins localize at the base of microtentacles. (**A**) Live cell confocal images of SEPT2-GFP transfected LNCaP, H1299 and Capan-2 cells. After detachment, cells were stained with CellMask Orange. Insets show the presence of SEPT2 at the base of microtentacles (arrows). (**B**) DIC images of MDA-MB-436 cells. Cells were left untreated or treated with CDT (200 ng/mL CDTa and 400 ng/mL CDTb) for 1 h before detachment and during image acquisition. (Scale bars, 5 μm)

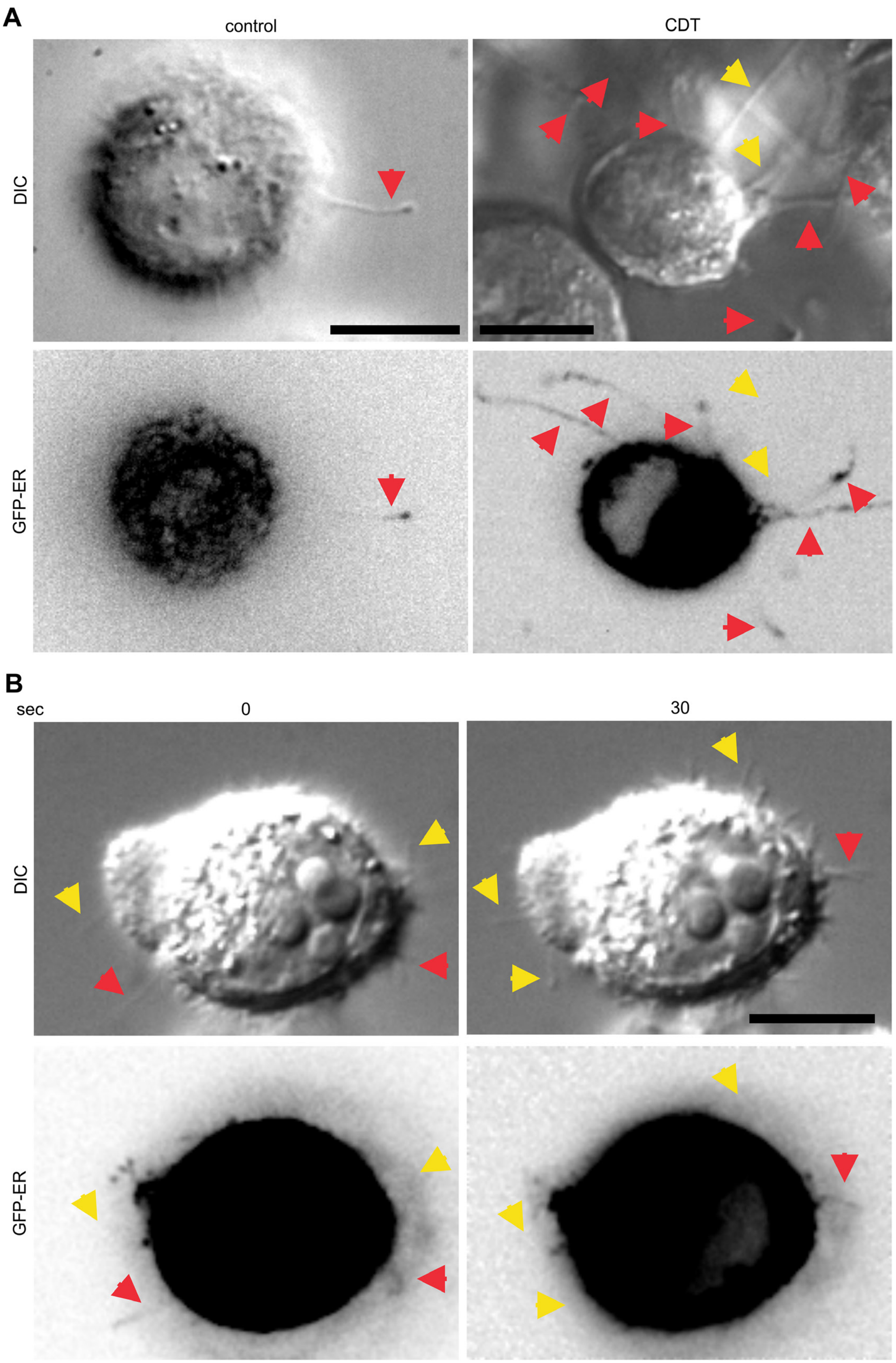

Supplementary Figure 3: ER is translocated to microtentacles. (**A**) DIC and confocal images of ER-GFP transfected MDA-MB-436 cells after detachment. Cells were left untreated or treated with CDT (200 ng/mL CDTa and 400 ng/mL CDTb) for 1 h prior to detachment and during image acquisition. ER can be seen in some (red arrows), but not all (yellow arrows) long microtentacles. (**B**) Cells treated as in A. Also here, ER can be seen in some (red arrows), but not all (yellow arrows) shorter protrusions. (Scale bars, 10 µm)

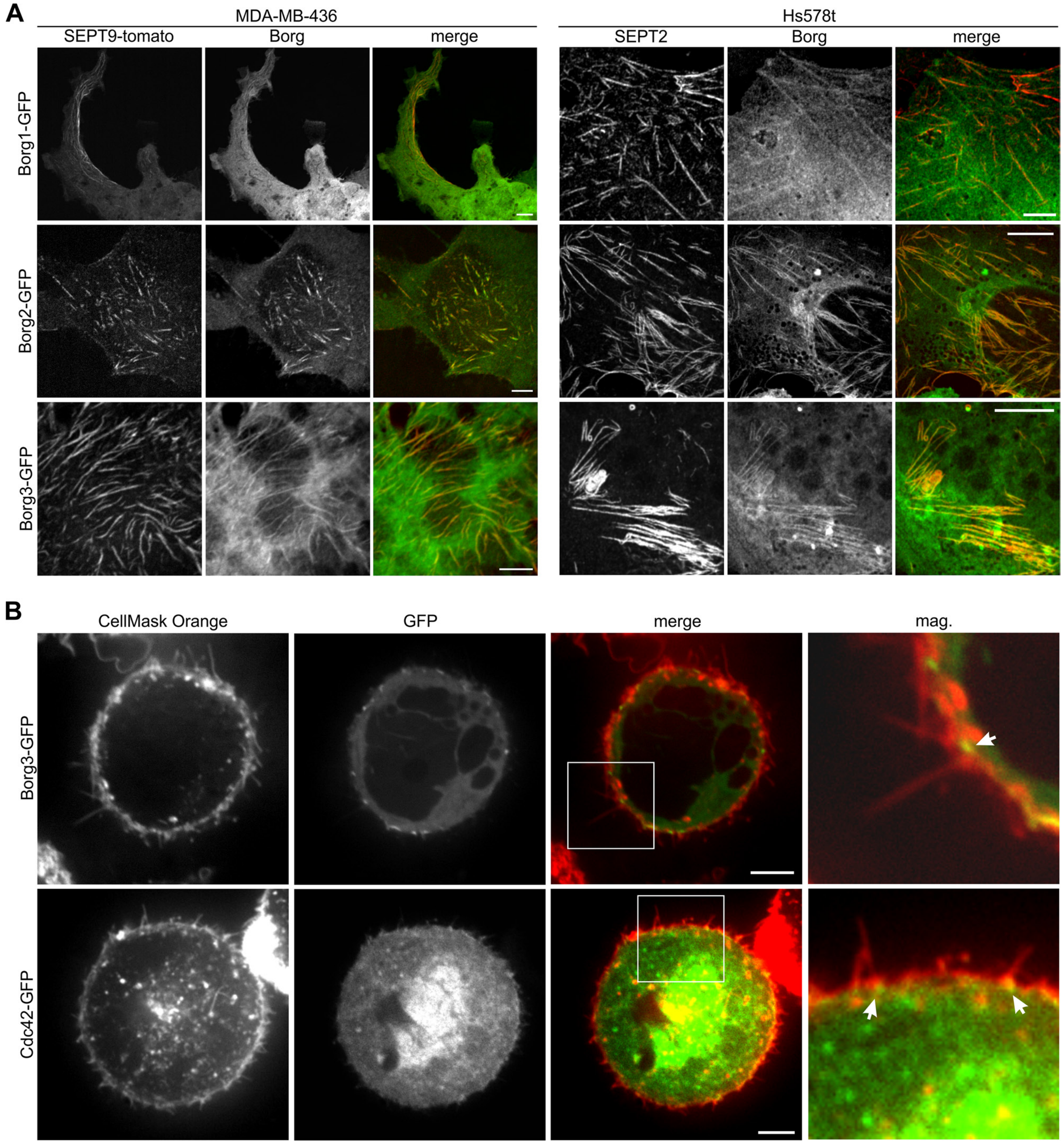

Supplementary Figure 4: Cdc42 and Borgs regulate septins during the formation of microtentacles. (**A**) Confocal images of MDA-MB-436 cells transfected with Borg 1-, 2-, or 3-GFP and SEPT9-tomato and indirect immunofluorescence for SEPT2 of Hs578t cells transfected with Borg 1-, 2-, or 3-GFP, Septin filaments show colocalization with Borgs. (**B**) Live cell confocal images of Borg3-GFP or Cdc42-GFP transfected Hs578t cells. After detachment, cells were stained with CellMask Orange. Insets show Borg3 and Cdc42 at the base of microtentacles (arrows). (Scale bars, 5 µm)

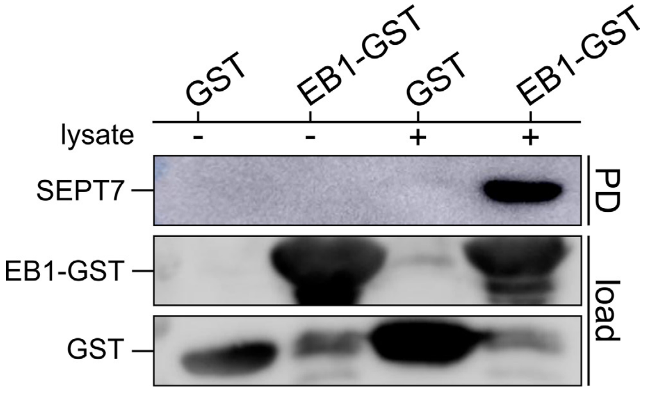

Supplementary Figure 5: Septins interact with EB1 in cancer cells. Representative blot of an EB1-GST pull-down of endogenous SEPT7 from MDA-MB-436 lysate. GST-loaded beads were used as a control.

Supplementary Movie 1: Septin rearrangement upon detachment of Hs578t cells. See Supplementary_Movie_1

Supplementary Movie 2: ER translocation after CDT treatment of detached MDA-MB-436 cells. See Supplementary_Movie_2

Supplementary Movie 3: ER translocation in detached MDA-MB-436 cells. See Supplementary_Movie_3

Supplementary Movie 4: Septin and Borg rearrangement upon detachment of Hs578t cells. See Supplementary_Movie_4

Supplementary Movie 5: Cell aggregation of MDA-MB-436 cells in hanging drop. See Supplementary_Movie_5
